# A case study of the informative value of risk of bias and reporting quality assessments for systematic reviews

**DOI:** 10.1186/s13643-024-02650-w

**Published:** 2024-09-07

**Authors:** Cathalijn H. C. Leenaars, Frans R. Stafleu, Christine Häger, André Bleich

**Affiliations:** 1 Institute for Laboratory Animal Science, Hannover Medical School, Carl Neubergstrasse 1, 30625 Hannover, Germany; 2 Department of Animals in Science and Society, Utrecht University, Yalelaan 2, Utrecht, 3584 CM the Netherlands

**Keywords:** Risk of bias, Reporting quality, Systematic reviews, Informative value, Cystic fibrosis, Nasal potential difference

## Abstract

While undisputedly important, and part of any systematic review (SR) by definition, evaluation of the risk of bias within the included studies is one of the most time-consuming parts of performing an SR. In this paper, we describe a case study comprising an extensive analysis of risk of bias (RoB) and reporting quality (RQ) assessment from a previously published review (CRD42021236047). It included both animal and human studies, and the included studies compared baseline diseased subjects with controls, assessed the effects of investigational treatments, or both. We compared RoB and RQ between the different types of included primary studies. We also assessed the “informative value” of each of the separate elements for meta-researchers, based on the notion that variation in reporting may be more interesting for the meta-researcher than consistently high/low or reported/non-reported scores. In general, reporting of experimental details was low. This resulted in frequent unclear risk-of-bias scores. We observed this both for animal and for human studies and both for disease-control comparisons and investigations of experimental treatments. Plots and explorative chi-square tests showed that reporting was slightly better for human studies of investigational treatments than for the other study types. With the evidence reported as is, risk-of-bias assessments for systematic reviews have low informative value other than repeatedly showing that reporting of experimental details needs to improve in all kinds of in vivo research. Particularly for reviews that do not directly inform treatment decisions, it could be efficient to perform a thorough but partial assessment of the quality of the included studies, either of a random subset of the included publications or of a subset of relatively informative elements, comprising, e.g. ethics evaluation, conflicts of interest statements, study limitations, baseline characteristics, and the unit of analysis. This publication suggests several potential procedures.

## Introduction

Researchers performing systematic reviews (SRs) face bias at two potential levels: first, at the level of the SR methods themselves, and second, at the level of the included primary studies [1]. To safeguard correct interpretation of the review’s results, transparency is required at both levels. For bias at the level of the SR methods, this is ensured by transparent reporting of the full SR methods, at least to the level of detail as required by the PRISMA statement [2]. For bias at the level of the included studies, study reporting quality (RQ) and/or risk of bias (RoB) are evaluated at the level of the individual included study. Specific tools are available to evaluate RoB in different study types [3]. Also, for reporting of primary studies, multiple guidelines and checklists are available to prevent missing important experimental details and more become available for different types of studies over time [4, 5]. Journal endorsement of these types of guidelines has been shown to improve study reporting quality [6].

While undisputedly important, evaluation of the RoB and/or RQ of the included studies is one of the most time-consuming parts of an SR. Experienced reviewers need 10 min to an hour to complete an individual RoB assessment [7], and every included study needs to be evaluated by two reviewers. Besides spending substantial amounts of time on RoB or RQ assessments, reviewers tend to become frustrated because of the scores frequently being unclear or not reported (personal experience from the authors, colleagues and students). While automation of RoB seems to be possible without loss of accuracy [8, 9], so far, this automation has not had significant impact on the speed; in a noninferiority randomised controlled trial of the effect of automation on person-time spent on RoB assessment, the confidence interval for the time saved ranged from − 5.20 to + 2.41 min [8].

In any scientific endeavour, there is a balance between reliability and speed; to guarantee reliability of a study, time investments are necessary. RoB or RQ assessment is generally considered to be an essential part of the systematic review process to warrant correct interpretation of the findings, but with so many studies scoring “unclear” or “not reported”, we wondered if all this time spent on RoB assessments is resulting in increased reliability of reviews.

Overall unclear risk of bias in the included primary studies is a conclusion of multiple reviews, and these assessments are useful in pinpointing problems in reporting, thereby potentially improving the quality of future publications of primary studies. However, the direct goal of most SRs is to answer a specific review question, and in that respect, unclear RoB/not reported RQ scores contribute little to the validity of the review’s results. If all included studies score “unclear” or “high” RoB on at least one of the analysed elements, the overall effect should be interpreted as inconclusive.

While it is challenging to properly evaluate the added validity value of a methodological step, we had data available allowing for an explorative case study to assess the informative value of various RoB and RQ elements in different types of studies. We previously performed an SR of the nasal potential difference (nPD) for cystic fibrosis (CF) in animals and humans, aiming to quantify the predictive value of animal models for people with CF [10, 11]. That review comprised between-subject comparisons of both baseline versus disease-control and treatment versus treatment control. For that review, we performed full RoB and RQ analyses. This resulted in data allowing for comparisons of RoB and RQ between animal and human studies, but also between baseline and treatment studies, which are both presented in this manuscript. RoB evaluations were based on the Cochrane collaboration’s tool [12] for human studies and SYRCLE’s tool [13] for animal studies. RQ was tested based on the ARRIVE guidelines [14] for animal studies and the 2010 CONSORT guidelines [15] for human studies. Brief descriptions of these tools are provided in Table 1.

**Table 1:** A brief description of the relevant reporting guidelines and risk-of-bias tools

Tool	Description
ARRIVE [14]	A 20-item checklist describing the information that all publications reporting animal research should include to correctly interpret the results
Cochrane’s [12]	The RoB 2 tool from the Cochrane collaboration provides a framework for assessing the risk of bias in a single result from a randomised clinical trial
CONSORT [15]	A checklist and suggested flow diagram that authors can use for reporting randomised clinical trials, created to aid critical appraisal and interpretation of the results
SYRCLE’s [13]	RoB tool for animal intervention studies, adjusted from the Cochrane tool for animal studies. Signalling questions were added to facilitate scoring

All these tools are focussed on interventional studies. Lacking more specific tools for baseline disease-control comparisons, we applied them as far as relevant for the baseline comparisons. We performed additional analyses on our RQ and RoB assessments to assess the amount of distinctive information gained from them.

## Methods

The analyses described in this manuscript are based on a case study SR of the nPD related to cystic fibrosis (CF). That review was preregistered on PROSPERO (CRD42021236047) on 5 March 2021 [16]. Part of the results were published previously [10]. The main review questions are answered in a manuscript that has more recently been published [11]. Both publications show a simple RoB plot corresponding to the publication-specific results.

For the ease of the reader, we provide a brief summary of the overall review methods. The full methods have been described in our posted protocol [16] and the earlier publications [10, 11]. Comprehensive searches were performed in PubMed and Embase, unrestricted for publication date or language, on 23 March 2021. Title-abstract screening and full-text screening were performed by two independent reviewers blinded to the other’s decision (FS and CL) using Rayyan [17]. We included animal and/or human studies describing nPD in CF patients and/or CF animal models. We restricted to between-subject comparisons, either CF versus healthy controls or experimental CF treatments versus CF controls. Reference lists of relevant reviews and included studies were screened (single level) for snowballing. Discrepancies were all resolved by discussions between the reviewers.

Data were extracted by two independent reviewers per reference in several distinct phases. Relevant to this manuscript, FS and CL extracted RoB and RQ data in Covidence [18], in two separate projects using the same list of 48 questions for studies assessing treatment effects and studies assessing CF-control differences. The *k* = 11 studies that were included in both parts of the overarching SR were included twice in the current data set, as RoB was separately scored for each comparison. Discrepancies were all resolved by discussions between the reviewers. In violation of the protocol, no third reviewer was involved.

RoB and SQ data extraction followed our review protocol, which states the following: “For human studies, risk of bias will be assessed with the Cochrane Collaboration’s tool for assessing risk of bias. For animal studies, risk of bias will be assessed with SYRCLE’s RoB tool. Besides, we will check compliance with the ARRIVE and CONSORT guidelines for reporting quality”. The four tools contain overlapping questions. To prevent unnecessary repetition of our own work, we created a single list of 48 items, which were ordered by topic for ease of extraction. For RoB, this list contains the same elements as the original tools, with the same response options (high/unclear/low RoB). For RQ, we created checklists with all elements as listed in the original tools, with the response options reported yes/no. For (RQ and RoB) elements specific to some of the included studies, the response option “irrelevant” was added. We combined these lists, only changing the order and merging duplicate elements. We do not intend this list to replace the individual tools; it was created for this specific study only.

In our list, each question was preceded by a short code indicating the tool it was derived from (A for ARRIVE, C for CONSORT, and S for SYRCLE’s) to aid later analyses. When setting up, we started with the animal-specific tools, with which the authors are more familiar. After preparing data extraction for those, we observed that all elements from the Cochrane tool had already been addressed. Therefore, this list was not explicit in our extractions. The extraction form always allowed free text to support the response. Our extraction list is provided with our supplementary data.

For RoB, the tools provide relatively clear suggestions for which level to score and when, with signalling questions and examples [12, 13]. However, this still leaves some room for interpretation, and while the signalling questions are very educative, there are situations where the response would in our opinion not correspond to the actual bias. The RQ tools have been developed as guidelines on what to report when writing a manuscript, and not as a tool to assess RQ [14, 15]. This means we had to operationalise upfront which level we would find sufficient to score “reported”. Our operationalisations and corrections of the tools are detailed in Table 2.

**Table 2:** Operationalisation of the analysed tools

Question in our template	Response options	Operationalisation
**ARRIVE** [19]		
A1a	Y/N	With Y, it is clear which groups were subject to specific experimental conditions
A1b	Y/N	With Y, it is clear what is used as a unit of analysis. If repeated measures were available, it needed to be clear how they were analysed, e.g. as separate repeated measures or as a mean value
A2a	Y/N	N per group and total are clear. If ranges were reported, we scored N
A2b	Y/N	Report of any a priori sample size was sufficient for a Y
A3a	Y/N	If potential criteria were described as demographic information only, we scored N
A3b	Y/N	If any exclusions were explicitly stated, we scored Y, even though there might have been more
^a^3c	-	Merged with A2a and A1b
^a^4a	-	Merged with S1
^a^4b	-	Merged with S4 and S6
a5	-	Merged with S3, S5, and S7
A6a	Y/N	All assessed outcome measures were explicitly mentioned for a Y
A6b	Y/N/I	The primary outcome measure, or that used for sample size calculation, was explicitly mentioned
A7a	Y/N/I	Methods AND software had to be mentioned for a Y
A7b	Y/N/I	Explicit or implicit (results only) mention of assumption testing resulted in a Y
^a^A8a	-	Merged with A9, C15, and S2
^a^A8b	-	Merged with A9, C15, and S2
A9a	Y/N	The extractors both had to feel sufficiently informed to initiate reproduction of the experiment. This comprised knowledge of the species, strain, age, sex/gender, health status, etc
A9b	Y/N	For a Y, information needed to be provided to know when the study was performed, up to the day of the week. E.g. “between June and September” [20] was scored N
A9c	Y/N	The laboratory needed to be clear for a Y, and for larger universities and hospitals with multiple laboratories, we scored N
A9d	Y/N	The rationale of at least two parts of the experimental design needed to be explained explicitly
^a^10a	-	Merged with A6b and A7a
^a^10b	-	Merged with A6b and A7a
^a^11	Y/N	N if any of the requested elements (mostly strain and sex) was missing from the abstract. For human studies, we ignored strain
^a^12a	Y/N	The extractors needed to understand the research question and its relevance for a Y
^a^12b	Y/N/I	The model validity had to be explicitly described for a Y. Not scored for human baseline studies
^a^13	Y/N	The objectives needed to be clear to both extractors for a Y
^a^14	Y/N	The registration number of the ethics proposal and the name of the committee needed to be provided for a Y
^a^15	Y/N/I	The extractors needed to have a reasonable idea of the inside of the cages for a Y. Mention of “standard” housing types could be sufficient. Not scored for human studies
^a^16a	Y/N/I	Any mention of refinement other than anaesthesia was sufficient for a Y. Not scored for human studies
^a^16b	Y/N	Any mention was sufficient for a Y
^a^16c	Y/N/I	Any mention was sufficient for a Y. Not scored for human studies
^a^17a	Y/N	Interpretation had to relate explicitly to theory/hypotheses/background literature/experimental set-up for a Y, and an overview of the results without interpretation resulted in an N
^a^17b	Y/N	Any explicit mention or at least two implicit mentions of limitations for a Y
^a^18	Y/N/I	Interpretation described the extent of external validity, either implicitly or explicitly, for a Y
^a^19	-	Merged with S9
^a^20	Y/N	We scored Y if the data were available where stated
^a^21a	Y/N	Y with explicit mention of conflicts or the absence thereof
^a^21b	Y/N	N if any of the requested elements (mostly involvement of the funder) was missing
**Cochrane’s RoB tool** [12]		
^a^1	-	Merged with C8b, C15, S1, and S2
^a^2	-	Merged with S2–S6
^a^3	-	Merged with A2A and S8
^a^4	-	Merged with S6 and S7
^a^5	-	Merged with S9
**CONSORT statement** [15]		
C1a	Y/N	Y if the title mentions the type of study. “Animal study” was not considered enough and “phenotypic model characterization” was
^a^1b	-	Merged with A11
^a^2a	-	Merged with A12a
^a^2b	-	Merged with A12b
C3a	Y/N	Y if the experimental study design was mentioned. Allocation ratio was merged with A2a
C3b	Y/N	Y if (absence of) protocol deviations were explicitly mentioned
^a^4a	Y/N	Merged with A3a
C4b	Y/N	More lenient than A9c; Y if we were certain of the type of settings (e.g. “laboratory” or “hospital”)
^a^5	-	Merged with A9a
^a^6a	-	Merged with A6b
^a^6b	-	Merged with A6a and C1a
^a^7a	-	Merged with A2b
C7b	Y/N/I	Any mention of interim analyses and/or stopping rules was sufficient for a Y. I for studies with a single measurement and explicitly short duration
^a^8a	-	Merged with S1
C8b	Y/N/I	The type of randomisation had to be mentioned. Irrelevant for studies without intervention
^a^9	-	Merged with S3
C10	Y/N	Y: It was clear who did what in group allocation. Irrelevant for studies without intervention
^a^11a	-	Merged with S5
^ a^11b	-	Merged with S5
^a^12a	-	Merged with A7a
C12b	Y/N/I	Y with minimal description of the methods for additional analyses. I for studies prespecifying a single analysis
^a^13a	-	Merged with A2a and S8
^a^13b	-	Merged with A2a and S8
^a^14a	-	Merged with A9b
^a^14b	-	Merged with C7b
C15	Y/N	For a Y, baseline data needed to be provided at group level for at least age/weight and genetics
^***a***^16	-	Merged with A2a
^a^17a	-	Merged with A10a and A10b
^a^17b	-	Merged with A10a and A10b
^a^18	-	Merged with C12b
^a^19	-	Merged with A16b
^a^20	-	Merged with A17b
^a^21	-	Merged with A18
^a^22	-	Merged with A17a
^a^23	-	Merged with S9
^a^24	-	Merged with S9
^a^25	-	Merged with A21b
**SYRCLE’s RoB tool** [13]		
S1	L/U/H/I	“Randomly picked from the box” would score U. I for studies without interventions. Allocation sequence generation was not scored for noninterventional studies
S2	L/U/H	For an L, baseline data needed to be comparable for at least age/weight, sex/gender, and type of mutation for intervention studies or genetic background for CF-control studies
S3	L/U/H/I	The investigator *allocating animals/participants* was adequately blinded for an L. Latin-square-like designs would always have scored an H. I for human studies without interventions. For animal model studies, we could have scored potential bias for the model generation here (but in the rare cases where it was relevant, it was U)
S4	L/U/H/I	Was there RoB related to the animal housing? In theory, Latin-square-like cage placement would have scored an L here. I for human studies
S5	L/U/H	The *investigators performing and/or caring for subjects during and between the experiments* were all adequately blinded for an L
S6	L/U/H	Was there RoB related to the outcome assessment (order/method)? In theory, counterbalanced orders would have scored an L here
S7	L/U/H	The *outcome assessors* were all adequately blinded for an L
S8	L/U/H	For an L-score, the data had to be either explicitly complete, or incomplete outcomes had to be equally distributed over the groups
S9	L/U/H	We were more strict than the tool here and always scored H if no protocol was posted
S10	L/U/H	We scored H if the methods were unclear or caused reasons for concern at points not addressed in any of the other elements

*Y* yes-reported, *N* no, not reported, *I* irrelevant, *H* high RoB, *U* unclear RoB, *L* low RoB

^a^Not numbered in our extraction template. Our data extraction template, with 48 questions, is provided in the supplementary file (first tab). The first letter in the question number always refers to the source of the question (A for ARRIVE, C for CONSORT, S for SYRCLE’s RoB tool). “Equally” refers to a difference of less than 5%

### Analysis

Data were exported from Covidence into Microsoft’s Excel, where the two projects were merged and spelling and capitalisation were harmonised. Subsequent analyses were performed in R [21] version 4.3.1 (“Beagle Scouts”) via RStudio [22], using the following packages: readxl [23], dplyr [24], tidyr [25], ggplot2 [26], and crosstable [27].

Separate analyses were performed for RQ (with two levels per element) and RoB (with three levels per element). For both RoB and RQ, we first counted the numbers of irrelevant scores overall and per item. Next, irrelevant scores were deleted from further analyses. We then ranked the items by percentages for reported/not reported, or for high/unclear/low scores, and reported the top and bottom 3 (RoB) or 5 (RQ) elements.

While 100% reported is most informative to understand what actually happened in the included studies, if all authors continuously report a specific element, *scoring* of this element for an SR is not the most informative for meta-researchers. If an element is not reported at all, this is bad news for the overall level of confidence in an SR, but evaluating it per included study is also not too efficient except for highlighting problems in reporting, which may help to improve the quality of future (publications of) primary studies. For meta-researchers, elements with *variation* in reporting may be considered most interesting because these elements highlight differences between the included studies. Subgroup analyses based on specific RQ/RoB scores can help to estimate the effects of specific types of bias on the overall effect size observed in meta-analyses, as has been done for example randomisation and blinding [28]. However, these types of subgroup analyses are only possible if there is some variation in the reporting. Based on this idea, we defined a “distinctive informative value” (DIV) for RQ elements, based on the optimal variation being 50% reported and either 0% or 100% reporting being minimally informative. Thus, this “DIV” was calculated as follows:

$$\mathrm{DIV}\;=\;\lbrack50\;-\;(\mathrm{distance}\;\mathrm{of}\;\%\mathrm Y\;\mathrm{to}\;50\%)\rbrack$$

$$\mathrm{With}\;\%\mathrm Y\;=\;\%\;\mathrm{reported}$$

**Fig. 1:**
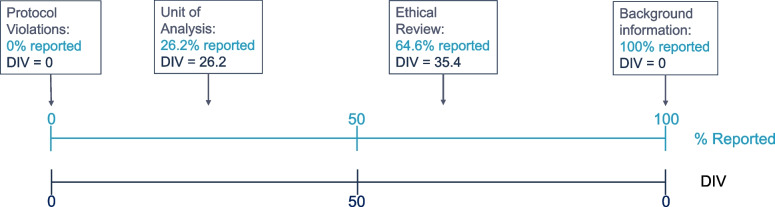
Visual explanation of the DIV value

Thus, the DIV could range from 0 (no informative value) to 50 (maximally informative), visualised in Fig. 1.

The DIV value was only used for ranking. The results were visualised in a heatmap, in which the intermediate shades correspond to high DIV values.

For RoB, no comparable measure was calculated. With only 10 elements but at 3 distinct levels, we thought a comparable measure would sooner hinder interpretation of informative value than help it. Instead, we show the results in an RoB plot split by population and study design type.

Because we are interested in quantifying the predictive value of animal models for human patients, we commonly perform SRs including both animal and human data (e.g. [29, 30]). The dataset described in the current manuscript contained baseline and intervention studies in animals and humans. Because animal studies are often held responsible for the reproducibility crisis, but also to increase the external validity of this work, explorative chi-square tests (the standard statistical test for comparing percentages for binary variables) were performed to compare RQ and RoB between animal and human studies and between studies comparing baselines and treatment effects. They were performed with the base R “chisq.test” function. No power calculations were performed, as these analyses were not planned.

## Results

### Literature sample

We extracted RoB and RQ data from 164 studies that were described in 151 manuscripts. These manuscripts were published from 1981 through 2020. Overall, 164 studies comprised 78 animal studies and 86 human studies, 130 comparisons of CF versus non-CF control, and 34 studies assessing experimental treatments. These numbers are detailed in a crosstable (Table 3).

**Table 3:** Cross-tabulation of included comparisons

	**Animals**	**Humans**	**Total**
Baseline CF-control	56	74	130
Treatment effects	22	12	34
Total	78	86	164

The 48 elements in our template were completed for these 164 studies, which results in 7872 assessed elements. In total, 954 elements (12.1%) were irrelevant for various reasons (mainly for noninterventional studies and for human studies). The 7872 individual scores per study are available from the data file on OSF.

Of the 48 questions in our extraction template, 38 addressed RQ, and 10 addressed RoB.

### Overall reporting quality

Of the 6232 elements related to RQ, 611 (9.8%) were deemed irrelevant. Of the remainder, 1493 (26.6% of 5621) were reported. The most reported elements were background of the research question (100% reported), objectives (98.8% reported), interpretation of the results (98.2% reported), generalisability (86.0% reported), and the experimental groups (83.5% reported). The least-reported elements were protocol violations, interim analyses + stopping rules and when the experiments were performed (all 0% reported), where the experiments were performed (0.6% reported), and all assessed outcome measures (1.2% reported).

The elements with most distinctive variation in reporting (highest DIV, refer to the “Methods” section for further information) were as follows: ethics evaluation (64.6% reported), conflicts of interest (34.8% reported), study limitations (29.3% reported), baseline characteristics (26.2% reported), and the unit of analysis (26.2% reported). RQ elements with DIV values over 10 are shown in Table 4.

**Table 4:** Distinctive informative values of at least 10 within the current sample

Reporting element	Percentage reported	DIV
Ethical review	64.6	35.4
Conflicts of interest	34.8	34.8
Limitations	29.3	29.3
Baseline values	26.2	26.2
Unit of analysis	26.2	26.2
Animal model relevance	74.4	25.6
Statistical methods	23.9	23.9
Number of animals (incl. humans)	81.1	18.9
Experimental groups	83.5	16.5
Methods — what was done?	15.9	15.9
Inclusion criteria	15.2	15.2
Generalisability	86.0	14.0
Housing and husbandry (animal studies)	12.8	12.8
Statistical assumption tests	12.3	12.3
Type of experimental design	12.2	12.2
Adverse events	10.4	10.4

### Overall risk of *bias*

Of the 1640 elements related to RoB, 343 (20.9%) were deemed irrelevant. Of the remainder, 219 (16.9%) scored high RoB, and 68 (5.2%) scored low RoB. The overall RoB scores were highest for selective outcome reporting (97.6% high), baseline group differences (19.5% high), and other biases (9.8% high); lowest for blinding of participants, caregivers, and investigators (13.4% low); blinding of outcome assessors (11.6% low) and baseline group differences (8.5% low); and most unclear for bias due to animal housing (100% unclear), detection bias due to the order of outcome measurements (99.4% unclear), and selection bias in sequence generation (97.1% unclear). The baseline group differences being both in the highest and the lowest RoB score are explained by the baseline values being reported better than the other measures, resulting in fewer unclear scores.

Variation in reporting is relatively high for most of the elements scoring high or low. Overall distinctive value of the RoB elements is low, with most scores being unclear (or, for selective outcome reporting, most scores being high).

### Animal versus human studies

**Fig. 2:**
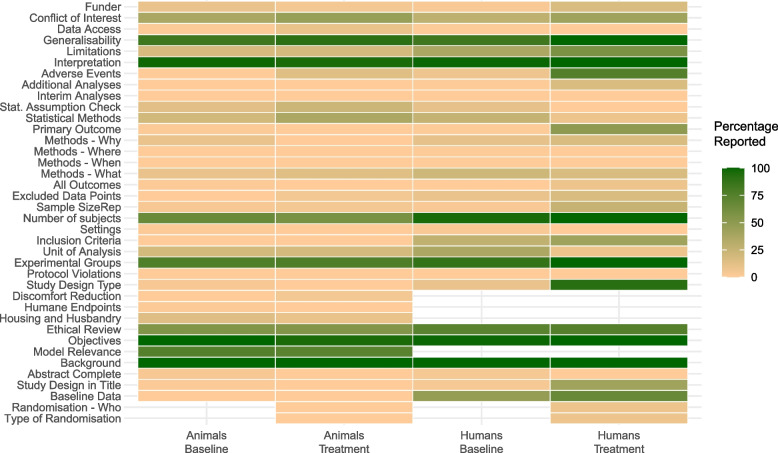
Heatmap of reporting by type of study. Refer to Table 3 for absolute numbers of studies per category

For RQ, the explorative chi-square tests indicated differences in reporting between animal and human studies for baseline values (*Χ*_1_ = 50.3, *p* < 0.001), ethical review (*Χ*_1_ = 5.1, *p* = 0.02), type of study (*Χ*_1_ = 11.2, *p* < 0.001), experimental groups (*Χ*_1_ = 3.9, *p* = 0.050), inclusion criteria (*Χ*_1_ = 24.6, *p* < 0.001), the exact n value per group and in total (*Χ*_1_ = 26.0, *p* < 0.001), (absence of) excluded datapoints (*Χ*_1_ = 4.5, *p* = 0.03), adverse events (*Χ*_1_ = 5.5, *p* = 0.02), and study limitations (*Χ*_1_ = 8.2, *p* = 0.004). These explorative findings are visualised in a heatmap (Fig. 2).

**Fig. 3:**
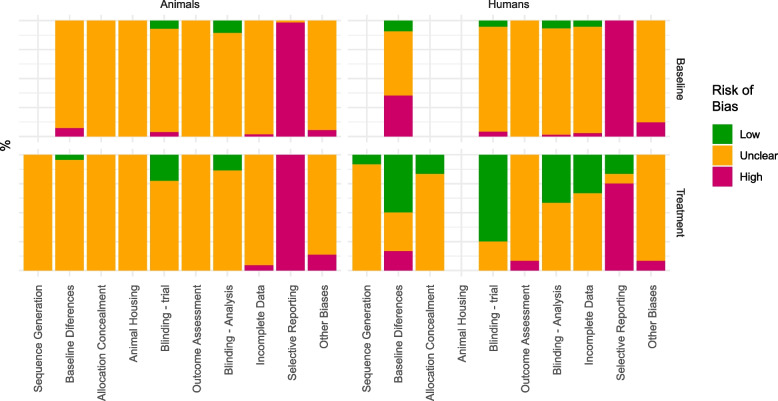
Risk of bias by type of study. Refer to Table 3 for absolute numbers of studies per category. Note that the data shown in these plots overlap with those in the two preceding publications [10, 11]

For RoB, the explorative chi-square tests indicated differences in risk of bias between animal and human studies for baseline differences between the groups (*Χ*_2_ = 34.6, *p* < 0.001) and incomplete outcome data (*Χ*_2_ = 7.6, *p* = 0.02). These explorative findings are visualised in Fig. 3.

### Studies assessing treatment effects versus studies assessing baseline differences

For RQ, the explorative chi-square tests indicated differences in reporting between comparisons of disease with control versus comparisons of treatment effects for the title listing the type of study (*X*_1_ = 5.0, *p* = 0.03), the full paper explicitly mentioning the type of study (*X*_1_ = 14.0, *p* < 0.001), explicit reporting of the primary outcome (*X*_1_ = 11.7, *p* < 0.001), and reporting of adverse events *X*_1_ = 25.4, *p* < 0.001). These explorative findings are visualised in Fig. 2.

For RoB, the explorative chi-square tests indicated differences in risk of bias between comparisons of disease with control versus comparisons of treatment effects for baseline differences between the groups (*Χ*_2_ = 11.4, *p* = 0.003), blinding of investigators and caretakers (*Χ*_2_ = 29.1, *p* < 0.001), blinding of outcome assessors (*Χ*_2_ = 6.2, *p* = 0.046), and selective outcome reporting (*Χ*_2_ = 8.9, *p* = 0.01). These explorative findings are visualised in Fig. 3.

Overall, our results suggest lower RoB and higher RQ for human treatment studies compared to the other study types.

## Discussion

This literature study shows that reporting of experimental details is low, frequently resulting in unclear risk-of-bias assessments. We observed this both for animal and for human studies, with two main study designs: disease-control comparisons and, in a smaller sample, investigations of experimental treatments. Overall reporting is slightly better for elements that contribute to the “story” of a publication, such as the background of the research question, interpretation of the results and generalisability, and worst for experimental details that relate to differences between what was planned and what was actually done, such as protocol violations, interim analyses, and assessed outcome measures. The latter also results in overall high RoB scores for selective outcome reporting.

Of note, we scored this more stringently than SYRCLE’s RoB tool [13] suggests and always scored a high RoB if no protocol was posted, because only comparing the “Methods” and “Results” sections within a publication would, in our opinion, result in an overly optimistic view. Within this sample, only human treatment studies reported posting protocols upfront [31, 32]. In contrast to selective outcome reporting, we would have scored selection, performance, and detection bias due to sequence generation more liberally for counterbalanced designs (Table 2), because randomisation is not the only appropriate method for preventing these types of bias. Particularly when blinding is not possible, counterbalancing [33, 34] and Latin-square like designs [35] can decrease these biases, while randomisation would risk imbalance between groups due to “randomisation failure” [36, 37]. We would have scored high risk of bias for blinding for these types of designs, because of increased sequence predictability. However, in practice, we did not include any studies reporting Latin-square-like or other counterbalancing designs.

One of the “non-story” elements that is reported relatively well, particularly for human treatment studies, is the blinding of participants, investigators, and caretakers. This might relate to scientists being more aware of potential bias of participants; they may consider themselves to be more objective than the general population, while the risk of influencing patients could be considered more relevant.

The main strength of this work is that it is a full formal analysis of RoB and RQ in different study types: animal and human, baseline comparisons, and treatment studies. The main limitation is that it is a single case study from a specific topic: the nPD test in CF. The results shown in this paper are not necessarily valid for other fields, particularly as we hypothesise that differences in scientific practice between medical fields relate to differences in translational success [38]. Thus, it is worth to investigate field-specific informative values before selecting which elements to score and analyse in detail.

Our comparisons of different study and population types show lower RoB and higher RQ for human treatment studies compared to the other study types for certain elements. Concerning RQ, the effects were most pronounced for the type of experimental design being explicitly mentioned and the reporting of adverse events. Concerning RoB, the effects were most pronounced for baseline differences between the groups, blinding of investigators and caretakers, and selective outcome reporting. Note, however, that the number of included treatment studies is a lot lower than the number of included baseline studies, and that the comparisons were based on only *k* = 12 human treatment studies. Refer to Table 3 for absolute numbers of studies per category. Besides, our comparisons may be confounded to some extent by the publication date. The nPD was originally developed for human diagnostics [39, 40], and animal studies only started to be reported at a later date [41]. Also, the use of the nPD as an outcome in (pre)clinical trials of investigational treatments originated at a later date [42, 43].

Because we did not collect our data to assess time effects, we did not formally analyse them. However, we had an informal look at the publication dates by RoB score for blinding of the investigators and caretakers, and by RQ score for ethics evaluation (in box plots with dot overlay), showing more reported and fewer unclear scores in the more recent publications (data not shown). While we thus cannot rule out confounding of our results by publication date, the results are suggestive of mildly improved reporting of experimental details over time.

This study is a formal comparison of RoB and RQ scoring for two main study types (baseline comparisons and investigational treatment studies), for both animals and humans. Performing these comparisons within the context of a single SR [16] resulted in a small, but relatively homogeneous sample of primary studies about the nPD in relation to CF. On conferences and from colleagues in the animal SR field, we heard that reporting would be worse for animal than for human studies. Our comparisons allowed us to show that particularly for baseline comparisons of the nPD in CF versus control, this is not the case.

The analysed tools [12, 13, 15] were developed for experimental interventional studies. While some of the elements are less appropriate for other types of studies, such as animal model comparisons, our results show that many of the elements can be used and could still be useful, particularly if the reporting quality of the included studies would be better.

### Implications

To correctly interpret the findings of a meta-analysis, awareness of the RoB in the included studies is more relevant than the RQ on its own. However, it is impossible to evaluate the RoB if the experimental details have not been reported, resulting in many unclear scores. With at least one unclear or high RoB score per included study, the overall conclusions of the review become inconclusive. For SRs of overall treatment effects that are performed to inform evidence-based treatment guidelines, RoB analyses remain crucial, even though the scores will often be unclear. Ideally, especially for SRs that will be used to plan future experiments/develop treatment guidelines, analyses should only include those studies consistently showing low risk of bias (i.e. low risk on *all* elements). However, in practice, consistently low RoB studies in our included literature samples (> 20 SRs to date) are too scarce for meaningful analyses. For other types of reviews, we think it is time to consider if complete RoB assessment is the most efficient use of limited resources. While these assessments regularly show problems in reporting, which may help to improve the quality of future primary studies, the unclear scores do not contribute much to understanding the effects observed in meta-analyses.

With PubMed already indexing nearly 300,000 mentioning the term “systematic review” in the title, abstract, or keywords, we can assume that many scientists are spending substantial amounts of time and resources on RoB and RQ assessments. Particularly for larger reviews, it could be worthwhile to restrict RoB assessment to either a random subset of the included publications or a subset of relatively informative elements. Even a combination of these two strategies may be sufficiently informative if the results of the review are not directly used to guide treatment decisions. The subset could give a reasonable indication of the overall level of evidence of the SR while saving resources. Different suggested procedures are provided in Table 5. The authors of this work would probably have changed to such a strategy during their early data extraction phase, if the funder would not have stipulated full RoB assessment in their funding conditions.

**Table 5:** Examples of potential SR procedures to evaluate the included studies and when to use them

Specific interest in RQ and/or RoB	Study sampling^a^	Tool/elements	Procedure
Yes, SR results will be used to plan future experiments/treatment guidelines	100%	Cochrane/SYRCLE	Full RoB analysis
Yes, to show the importance and effect of reporting measures on an outcome	100%	Check ARRIVE/CONSORT	Analysis of selected elements with high DIV value
Only to see if reporting in my field is different from other fields	Random: 5–50%	Check ARRIVE/CONSORT	Analysis of selected elements with high DIV value
No but interested in all aspects of study quality	Random: 5–50%	Cochrane/SYRCLE/Check ARRIVE/CONSORT	Full RoB/RQ analysis of a subset of the included studies to get an overall crude idea of the level of evidence in all aspects
No and only interested in specific aspects of study quality	Random: 25–50%	Cochrane/SYRCLE/Check ARRIVE/CONSORT	Analysis of selected elements with high DIV value

^a^For sampling, the total sample size is a relevant factor. It is important to sample, e.g. 50% for reviews that include ≤ 50 papers in total, assessing RoB/RQ in at least 25 of them, while 5% results in an informative sample for reviews including ≥ 1000 primary studies

We previously created a brief and simple taxonomy of systematised review types [44], in which we advocate RoB assessments to be a mandatory part of any SR. We would still urge anyone calling their review “systematic” to stick to this definition and perform some kind of RoB and/or RQ assessment, but two independent scientists following a lengthy and complex tool for all included publications, resulting in 74.6% of the assessed elements not being reported, or 77.9% unclear RoB, can, in our opinion, in most cases be considered inefficient and unnecessary.

## Conclusion

Our results show that there is plenty of room for improvement in the reporting of experimental details in medical scientific literature, both for animal and for human studies. With the current status of the primary literature as it is, full RoB assessment may not be the most efficient use of limited resources, particularly for SRs that are not directly used as the basis for treatment guidelines or future experiments.

## Data Availability

The data described in this study are available from the Open Science Platform (https://osf.io/fmhcq/) in the form of a spreadsheet file. In the data file, the first tab shows the list of questions that were used for data extraction with their respective short codes. The second tab shows the full individual study-level scores, with lines per study and columns per short code.

## Abbreviations

CF: Cystic fibrosis
H: High risk of bias
I: Irrelevant
L: Low risk of bias
N: No, not reported
nPD: Nasal potential difference
RoB: Risk of bias
RQ: Reporting quality
SR: Systematic review
U: Unclear risk of bias
Y: Yes, reported

## Declarations

Ethics approval and consent to participate.

Not applicable.
